# Linking neuroinflammation and neurodegeneration to cognitive decline in HIV

**DOI:** 10.1016/j.bbih.2026.101241

**Published:** 2026-04-14

**Authors:** Ronald J. Ellis, Yajing Bao, Huichao Chen, Scott Letendre, Ahmed Chenna, Brandon Yee, John Winslow, Christos Petropoulos, Shelli Farhadian, Shibani S. Mukerji, Albert Anderson

**Affiliations:** a Departments of Neurosciences and Psychiatry, University of California, San Diego, United States; b Statistical and Data Analysis Center (SDAC), Center for Biostatistics in AIDS Research (CBAR), Harvard T.H. Chan School of Public Health, Boston, MA, United States; c Departments of Medicine and Psychiatry, University of California, San Diego, United States; d Monogram Biosciences, Inc. (A Wholly-Owned Subsidiary of Labcorp Holdings, Inc.), South San Francisco, CA, United States; e Department of Medicine (Infectious Diseases), Yale School of Medicine, New Haven, CT, United States; f Department of Neurology, Massachusetts General Hospital and Harvard Medical School, Boston, MA, United States; g Department of Medicine, Division of Infectious Diseases, Emory University, Atlanta, GA, United States

## Abstract

**Background and objectives:**

We investigated the relationship between cerebrospinal fluid (CSF) and plasma biomarkers of inflammation, neurodegeneration, and neurocognitive performance in people with HIV (PWH), using longitudinal samples from two previously published cohorts: ACTG A5090 (virally suppressed on antiretroviral therapy, ART) and A736 (ART-naïve or failing).

**Methods:**

We analyzed paired CSF and plasma samples, as well as 7-domain standardized neurocognitive test scores, at baseline and 24 weeks. Biomarkers included markers of inflammation (e.g., TNF-α, IL-6, IP-10) and neurodegeneration (e.g., NFL, p-Tau217, Aβ42), which were quantified via high-sensitivity immunoassays. Associations with cognition were tested using regression, mediation, and interaction models.

**Results:**

Cross-sectional analyses revealed nominal associations between inflammatory markers and cognitive performance, with plasma IL-6 and IP-10 at baseline, and CSF TNFα at week 24 showing the strongest correlations (p < 0.05, uncorrected); however, none survived correction for multiple comparisons. Conversely, higher CSF Aβ42 and plasma BDNF were positively associated with memory and executive function. Longitudinally, biomarker changes did not significantly predict change in global cognition (ΔNPZ-8); the strongest trend (p-Tau217, ρ = −0.12, p = 0.38) was not statistically significant, and multivariate models failed to identify robust predictors (R^2^ < 0.15). Exploratory mediation analysis suggested CSF TNFα, but not plasma TNFα, partially mediated the effect of CSF HIV RNA on cognition (indirect β = 0.14, 95% CI: 0.045-0.235, p = 0.006), though this finding requires replication in larger cohorts. Within-compartment biomarker correlations were stronger in CSF than plasma, and cross-compartment agreement was highest for TNFα, GFAP, and NFL. ART initiation in A736 led to significant declines in CSF IL-6, IL-10, and TNFα; no changes were observed in A5090. Exploratory interaction models suggested that astrocytic activation may amplify tau-related cognitive risk, but these effects were not statistically reliable when analyses were restricted to participants with complete data for GFAP, tau biomarkers, and cognitive scores.

**Discussion:**

These results suggest a potential role of CSF TNFα in mediating the neurocognitive effects of HIV and highlight compartment-specific inflammatory dynamics. Plasma TNFα, GFAP, and NFL may serve as peripheral indicators of CNS pathology, though with only moderate concordance. Astrocyte–tau interactions require cautious interpretation pending replication in larger cohorts.

## Highlights

• ART rapidly lowers key CSF inflammatory markers in untreated HIV.
• Preliminary evidence suggests CSF TNF-α may explain an association between HIV viral load and poorer cognitive performance.
• Plasma GFAP and NFL partially mirror CNS injury markers.
• Results reveal compartment-specific drivers of neuroHIV.

## Graphical abstract

**Figure:**
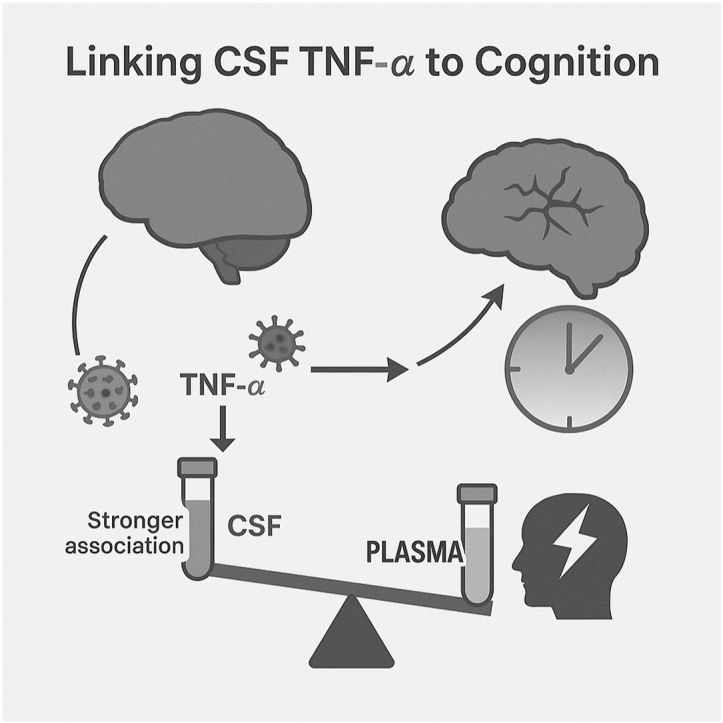

## 1 Introduction

People with HIV (PWH) display higher rates of cognitive impairment and of age-associated dementias, even when fully viral suppressed with ART (Wang et al., 2020; Gannon et al., 2011). Neurocognitive tests, while extremely useful, are difficult to perform in some real-world settings, and biomarkers of cognitive performance would help identify PWH in need of further services. Vascular comorbidity, chronic inflammation, substance use, and polypharmacy contribute to this burden (Smith et al., 2022; Wright et al., 2010), yet no biomarkers exist to distinguish the potential contributions of these factors to cognitive impairment (Gannon et al., 2011). Longitudinal multi-analyte studies directly comparing viremic and virologically suppressed cohorts are scarce; the predictive hierarchy of inflammatory and neurodegenerative markers has not been comprehensively mapped; plasma biomarkers that reliably mirror CSF pathology—glial fibrillary acidic protein (GFAP)—require validation; interactions between HIV-related astrocytic activation, tau phosphorylation, and age-related neurodegeneration remain poorly characterized (Ellis et al., 2022; Pereira et al., 2021; Thela et al., 2023; Pulliam et al., 2019; Mukerji et al., 2025); and no study has tested whether CSF rather than plasma tumour necrosis factor-α (TNF-α) transmits the cognitive impact of ongoing viral replication.

To close these gaps, we tracked longitudinal trajectories of inflammatory (GFAP, interleukin-6, TNF-α) and Alzheimer-related biomarkers (amyloid-β40/42, p-Tau181, p-Tau217, t-Tau), compared changes during ART initiation (A736) versus stable suppression (A5090), and examined compartment-specific biomarker–cognition associations and interactions. We included p-Tau217, omitted from prior work such as Li et al. (2023), to enhance clinical relevance. Because TNF-α plays a distinct role within the CNS compartment that is not mirrored in the periphery (Borrajo et al., 2021) (Malik and Eugenin, 2016), and because persistent HIV replication in the CNS drives local microglial and astrocytic activation, leading to increased CSF TNF-α (Chen et al., 1997), we hypothesized that CSF—but not plasma—TNF-α would mediate the effect of persistent HIV replication on cognition, and that elevated GFAP would amplify tau-related cognitive decline, particularly in PWH as they age. The overarching goal of this work was to strengthen the existing literature on the neuropathogenesis of cognitive impairment in people with HIV with longitudinal data from well-controlled interventional trials.

## 2 Methods

**Fig. 1:**
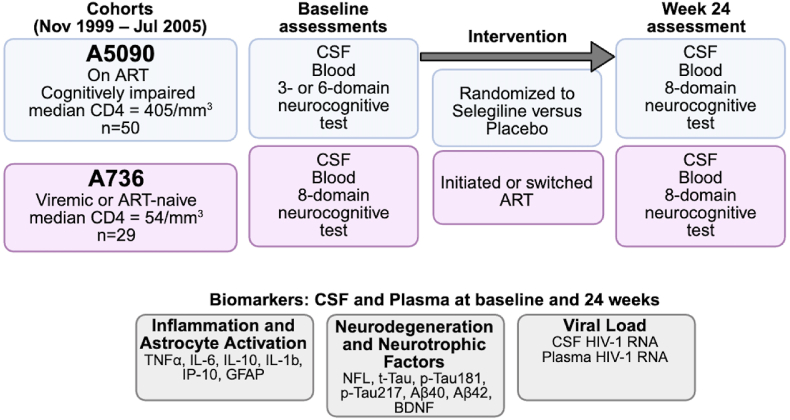
Study cohorts (see text).

Participants. We analyzed data generated using paired cerebrospinal fluid (CSF) and plasma samples from two ACTG cohorts: A5090 (n = 50) and A736 (n = 29), collected between November 1999 and July 2005. The primary studies reported total enrollment, whereas our analyses were limited to participants who met prespecified specimen and data requirements: paired CSF and plasma available at both baseline and Week 24 with adequate volume (about 2 mL, typically 2–4 aliquots) per specimen per visit; and complete neurocognitive data. Participants with only one visit, insufficient aliquots (often CSF; highlighted in yellow), or samples available only by combining screening/entry/pre-entry draws (highlighted in orange) were not included. These availability and completeness criteria explain why the analytic sample is smaller than the number reported in the parent studies. Participants were PWH recruited under distinct clinical criteria (Fig. 1). In ACTG A736, participants were either off antiretroviral therapy (ART) or experiencing virologic failure, with CD4^+^ T-cell counts <200 cells/mm^3^ and plasma HIV-1 RNA >2000 copies/mL, or HIV-1 RNA >50,000 copies/mL regardless of CD4 count. The participant's primary provider chose the new ART regimens. Enrollment did not require self-reported cognitive complaints, but all participants completed standardized neuropsychological testing. The ACTG A5090 cohort enrolled PWH on stable ART who met criteria for neurocognitive impairment based on formal testing; participants were randomized to placebo or two dose levels of selegiline, and samples were collected at screening and 24 weeks post-randomization. In both studies, neurocognitive status was determined through structured neuropsychological testing. Complete virologic suppression (<50c/mL in cell-free CSF) at baseline was uncommon, likely reflecting the era's reliance on protease inhibitor–based or efavirenz-based regimens in the setting of viral mutations that conferred resistance. All participants gave informed consent, and all specimens were de-identified and approved for use under original IRB protocols.

Biomarker quantification. Biomarkers were quantified using the Quanterix Simoa platform for high-sensitivity detection, including neurology markers (Aβ42, Aβ40, neurofilament light chain [NF-L], glial fibrillary acidic protein [GFAP], total tau [t-Tau], brain-derived neurotrophic factor [BDNF], phosphorylated tau 181 [p-Tau181]) and inflammation markers (tumor necrosis factor-alpha [TNFα], interleukin-6 [IL-6], IL-10, interferon gamma-induced protein 10 [IP-10], IL-1β). All biomarker assays were performed using standardized protocols on the Quanterix Simoa HD-X platform. Quality controls included duplicate measurements for each sample, with the average used for analysis. Intra-assay coefficients of variation were <8% for all analytes, and inter-assay CVs were <12%. Samples below the lower limit of detection were imputed as 50% of the detection limit. Laboratory personnel were blinded to clinical outcomes and study group assignments."

Neurocognitive (NC) testing**.** The previously-reported studies from which participants in the present study came assessed NC performance in both cohorts using two similar, standardized 6- to 7-domain test batteries (Supplemental Table 1). In ACTG A5090, all participants had documented impairment at study entry, with most classified as mild to moderate cognitive impairment. Scores were standardized to normative data from HIV-negative participants in the Multicenter AIDS Cohort Study (MACS). Cognitive impairment was defined as ≥1 standard deviation (SD) below normative means on at least two tests or ≥2 SD below on any single test. In ACTG A736, neurocognitive testing was performed at baseline and follow-up using an eight-test battery (NPZ-8), and age-adjusted Z-scores were calculated as in A5090. A736 enrolled participants regardless of the presence or absence of cognitive impairment; only a subset of A736 participants met criteria for impairment at entry. In both cohorts, lower composite Z-scores reflect poorer cognitive performance.

Statistical Analyses. Continuous variables were summarized as medians with interquartile ranges, and categorical variables as frequencies. Within-group changes from baseline to Week 24 were assessed using paired t-tests for normally distributed data and Wilcoxon signed-rank tests for non-normal data, while between-cohort comparisons used Welch's t-tests when variances differed and Wilcoxon rank-sum tests for non-normal distributions. Biomarker concentrations were analyzed on the log10 scale consistent with prior normalization, and cognitive change was defined as the square-root transformed difference in NPZ-8 from baseline to Week 24 to reduce skewness while preserving directionality. Log10 Aβ40, Aβ42, GFAP, NFL, t-Tau, p-Tau181, and p-Tau217 were analyzed with paired t-tests, whereas TNF-α, IL-6, IL-10, IP-10, IL-1β, and √ΔNPZ-8 were analyzed with Wilcoxon signed-rank tests because they remained non-normal after transformation.

*Missing Data and Sample Size*. Complete-case analyses were performed for primary models. Sensitivity analyses using multiple imputation (m = 10) were conducted for key findings to assess robustness to missing data assumptions.

*Multiple Comparisons and Statistical Significance*. Given the exploratory nature of this biomarker discovery analysis, we report both uncorrected p-values and false discovery rate (FDR)-corrected q-values using the Benjamini-Hochberg procedure. Primary significance was set at FDR q < 0.05, with nominal associations (p < 0.05, uncorrected) noted as trends requiring replication. For the 24 biomarkers tested across 3 time points, this approach controls family-wise error while preserving sensitivity for hypothesis generation.

*Data Visualization.* Spearman rank correlations were computed for biomarker-biomarker and biomarker-cognition associations at Weeks 0 and 24, chosen for robustness to outliers and minimal distributional assumptions. Correlation matrices used pairwise complete observations, were manually ordered by biological class, and visualized as grayscale heatmaps with uniform scaling. Cross-compartment plasma-CSF concordance was assessed by Pearson correlations of log10 concentrations using pairwise deletion for missing data. Within-subject biomarker changes used paired t-tests on log10-transformed values with results reported as p-values, Cohen's d, and 95% confidence intervals.

*Analysis of changes in neurocognitive performance.* Multivariable regression models examined NPZ-8 at baseline and Week 24 plus change scores, with individual biomarkers as predictors adjusted for age, sex, education, CD4 count, and plasma and CSF HIV RNA. All continuous predictors and outcomes were standardized so that coefficients represent effects per standard deviation. Biomarker-cognition associations were restricted to analytes with at least 10 non-missing values and displayed in volcano plots showing effect size versus significance, with points labeled if p < 0.1 and color-coded by fluid type. P-values were adjusted for multiple comparisons using Benjamini-Hochberg false discovery rate correction, with q less than 0.05 considered significant. Interaction models tested whether astrocytic inflammation modified associations between tau or amyloid biomarkers and cognition, modeling GFAP both continuously and by tertile with predictors log-transformed and standardized. Analyses included only participants with complete NPZ-8, GFAP, and target biomarker data. Exploratory combined biomarker models used standard linear regression to evaluate multi-marker panels.

*Mediation Analysis*. Mediation models were implemented using the 'mediation' package in R with bias-corrected bootstrap confidence intervals (5000 iterations). Indirect effects were considered significant if 95% confidence intervals excluded zero. These analyses were exploratory and not prespecified in the original study protocols.

All analyses were conducted in Python 3.10 and R 3.6.3, with visualization details provided in figure legends. The analytical approach prioritized prespecified hypotheses while acknowledging exploratory findings that require replication in larger cohorts.

## 3 Results

Participant characteristics (Table 1). The study included 79 participants from two clinically distinct HIV cohorts. The A5090 cohort (n = 50) consisted of ART-experienced individuals with partially controlled infection, preserved immune function (median CD4^+^ count 405 cells/mm^3^, IQR 236–574), and low viral loads in plasma (median 1.7 log_10_ copies/mL, IQR 1.7–2.8) and CSF (median 1.7, IQR 1.7–1.7). One participant in A5090 had missing plasma HIV RNA data at baseline but undetectable CSF HIV RNA (<50 co/mL); this individual was classified as virally suppressed based on CSF measures, bringing the total number of suppressed participants to 30. The A736 cohort (n = 29) comprised ART-naïve or failing individuals with uncontrolled infection, significant immunosuppression (median CD4^+^ count 54 cells/mm^3^, IQR 21–92), and high viral loads in plasma (median 4.8 log_10_ copies/mL, IQR 4.5–5.1) and CSF (median 3.6, IQR 2.4–3.9). Participants in the ACTG 736 cohort either initiated ART or changed their regimen due to virologic failure. Both cohorts were predominantly male (A5090 94.0%, A736 93.1%) with comparable education levels and racial distributions.

**Table 1:** Baseline Participant Demographics and Clinical Characteristics. Statistical comparisons between cohorts used Welch's t-tests for continuous variables and Fisher's exact tests for categorical variables. P-values were not adjusted for multiple comparisons given the descriptive nature of baseline characteristics. Values in bold were significantly different between the A5090 and A736 cohorts.

Characteristic	All (N = 79)	A5090 Cohort (N = 50)	A736 Cohort (N = 29)
Age, years, median [IQR]	45 [40–50]	**46 [42**–**51]**	**40 [34**–**46]**
Education, years, median [IQR]	13 [12–16]	14 [12–16]	13 [12–15.5]
Male, n (%)	74 (93.7%)	47 (94.0%)	27 (93.1%)
Race/Ethnicity, n (%)			
White non-Hispanic	38 (48.1%)	23 (46.0%)	15 (51.7%)
Black non-Hispanic	25 (31.6%)	17 (34.0%)	8 (27.6%)
Hispanic	7 (8.9%)	3 (6.0%)	4 (13.8%)
Asian/Pacific Islander	5 (6.3%)	4 (8.0%)	1 (3.4%)
American Indian/Alaskan Native	1 (1.3%)	1 (2.0%)	0 (0%)
Multiple races/Unknown	3 (3.8%)	2 (4.0%)	1 (3.4%)
Current CD4^+^ lymphocytes mm^3^, median [IQR]	207 [86–512]	405 [236–574]	**54 [21**–**92]**
Plasma HIV RNA (log_10_ c/mL), median [IQR]	3.1 [1.7–4.7]	**1.7 [1.7**–**2.8]**	**4.8 [4.5**–**5.1]**
Virally suppressed in plasma (<50c/mL)	27 (34.2%)	**26 (52%)**	**1∗ (0.34%)**
CSF HIV RNA (log_10_ copies/mL), median [IQR]	1.7 [1.7–3.5]	**1.7 [1.7**–**1.7]**	**3.6 [2.4**–**3.9]**
On ART, n (%)	50 (63.3%)	**50 (100%)**	**0 (0%)**
Baseline neurocognitive performance: NPZ-8	−0.539 [-1.10, −0.216]	**−0.745 [-1.33, -0.462]**	**−0.145 [-0.402, 0.249]**

∗One participant in A736 had missing plasma HIV RNA data at baseline but undetectable CSF HIV RNA (<50 co/mL). This individual was classified as virally suppressed based on CSF measures.

**Fig. 2:**
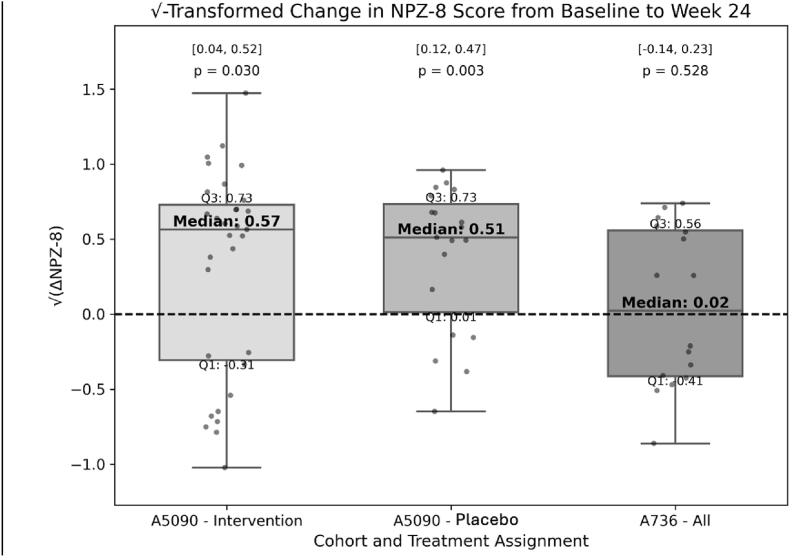
Change in square-root–transformed global neurocognitive performance (NPZ-8) from baseline to Week 24, stratified by cohort and treatment arm. Boxplots display square-root–transformed NPZ-8 change scores (√[ΔNPZ-8]) for A5090 Intervention (n = 31), A5090 Control (n = 19), and A736 (n = 29), with overlaid individual data points. Y-axis values reflect the transformed metric used in all statistical comparisons. Group medians and interquartile ranges (IQRs) are annotated. Wilcoxon signed-rank test p-values test whether each median change differs from zero; 95% confidence intervals for mean ΔNPZ-8 (untransformed) are also shown. Dashed line indicates no change.

Changes in neurocognitive performance by study. Square-root transformed NPZ-8 scores revealed significant cognitive improvement in both A5090 intervention and control arms (p = 0.030 and p = 0.003, respectively) with no between-arm differences, while the A736 cohort showed no significant change overall (p = 0.528) (Fig. 2). Baseline viral suppression did not predict differential cognitive gains. Among 29 virally suppressed participants, mean NPZ-8 change was 0.14 (SD = 0.43), while 36 non-suppressed participants improved by 0.30 (SD = 0.58), yielding a non-significant difference (t = −1.28, p = 0.21, Cohen's d = −0.31).

**Fig. 3:**
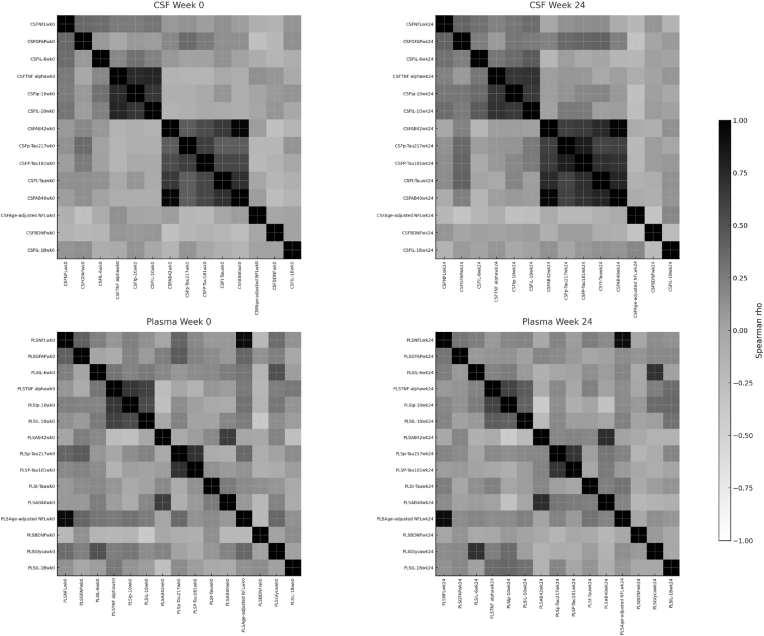
Within-compartment biomarker correlations in cerebrospinal fluid (CSF, top panels) and plasma (bottom panels) at baseline (Week 0, left) and Week 24 (right). Spearman correlation coefficients (ρ) were calculated and displayed after reordering variables into a fixed biologically relevant order that places inflammatory cytokines, glial injury, axonal injury, and amyloid markers together. Grayscale shading indicates correlation strength and direction (darker = stronger positive, mid-gray = near zero, white = negative). In CSF, this ordering highlights a tightly correlated module spanning IL-6, TNF-α, IP-10, GFAP, NFL, and amyloid/tau markers across both timepoints. Plasma correlations were weaker and less consistently modular.

Within-compartment biomarker inter-correlation patterns. Within-compartment biomarker networks analyzed using Spearman correlation revealed distinct patterns (Fig. 3). CSF markers exhibited strong positive correlations forming a persistent contiguous block, with 27.8% of pairs reaching FDR significance versus 19.4% in plasma. CSF Aβ40 and Aβ42 were nearly colinear (ρ = 0.97, p < 2.2e-44), while inflammatory markers IL-6, IL-10, IP-10, and TNF-α formed a tightly correlated module (ρ range 0.46–0.64) stable from baseline to Week 24. CSF total tau and GFAP were not significantly correlated (ρ = 0.14, p = 0.25). Plasma correlations were consistently weaker and more variable, with only NFL and GFAP showing a moderate association (ρ = 0.36, p = 0.0018). HIV RNA levels correlated significantly with inflammatory cytokines in both compartments. CSF HIV RNA showed a strong correlation with TNF-α (ρ = 0.73, p < 2 × 10^−12^) but a weaker association with IL-6 (ρ = 0.20, p = 0.11). Plasma HIV RNA correlated moderately with TNF-α (ρ = 0.70, p < 1.4 × 10^−11^) but not IL-6 (ρ = 0.07, p = 0.56). NFL showed moderate correlation with GFAP in CSF but minimal association with HIV RNA, suggesting partially independent neurodegenerative processes.

**Fig. 4:**
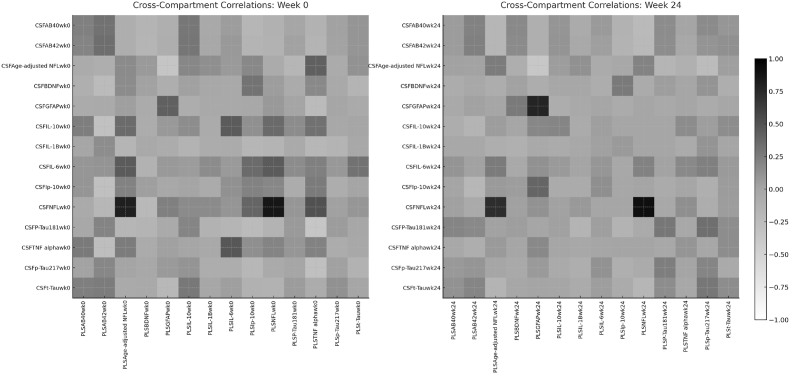
Cross-compartment correlations between plasma and cerebrospinal fluid (CSF) concentrations of matching biomarkers at baseline (Week 0, left) and Week 24 (right). Heatmaps show Pearson correlation coefficients (r) between log-transformed plasma and CSF values for each analyte. Grayscale shading indicates correlation strength and direction (darker = stronger positive, mid-gray = near zero, white = negative).

**Fig. 5:**
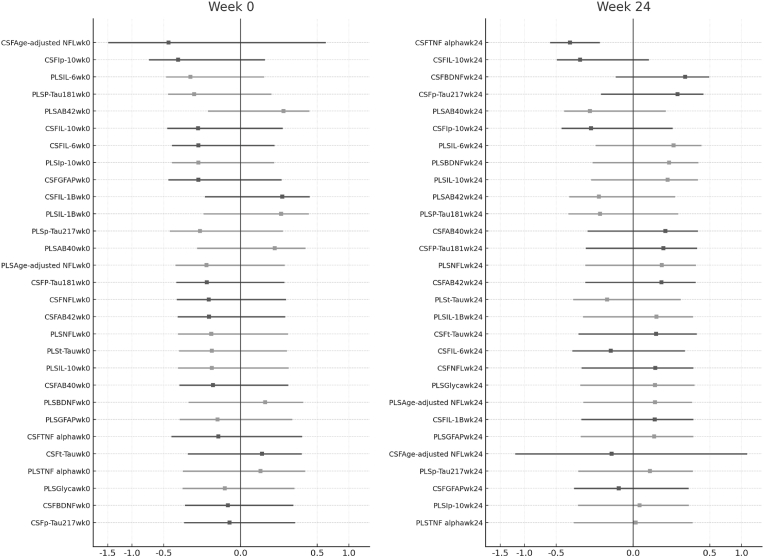
Interaction effects of BBB integrity and viral suppression on plasma–CSF biomarker associations. The left panel shows the top 15 interaction terms from models testing whether blood–brain barrier (BBB) impairment modified correlations between plasma and CSF biomarkers. The right panel shows the top 15 interaction terms from models testing whether viral suppression status modified plasma–CSF correlations (positive values indicate stronger associations in unsuppressed participants). Estimates are standardized regression coefficients (β) with 95% confidence intervals, derived from linear models adjusted for age, sex, education, CD4 count, and HIV RNA. Biomarkers are ordered by absolute effect size. A shared x-axis scale is used within each panel to allow direct comparison across analytes.

Cross-compartment biomarker concordance and relationship to viral suppression. As seen in the heatmap in Fig. 4, at baseline, several biomarkers showed moderate-to-strong plasma-CSF correlations, including NFL (ρ = 0.70), GFAP (ρ = 0.38), TNF-α (ρ = 0.44), IL-10 (ρ = 0.30), and Aβ42 (ρ = 0.31). By Week 24, most peripheral-central associations weakened, with only NFL (r = 0.90) and GFAP (r = 0.78) remaining FDR-significant, suggesting reduced coherence between compartments following treatment, while neuronal and astrocytic injury markers maintained stability. To understand factors influencing these plasma-CSF relationships, we examined whether blood-brain barrier integrity and viral suppression status modified biomarker concordance. Blood-brain barrier permeability, assessed using the CSF:plasma albumin ratio, showed limited influence on most correlations (Fig. 5), though several inflammatory markers demonstrated enhanced concordance with greater barrier disruption, particularly IL-6 (β = 0.24, 95% CI: 0.02-0.46, p = 0.032) and TNF-α (β = 0.19, 95% CI: −0.03-0.41, p = 0.089). Viral suppression status emerged as a more consistent modifier of cross-compartment relationships. Participants with detectable HIV RNA showed stronger plasma-CSF correlations for inflammatory markers compared to virally suppressed individuals, with TNF-α demonstrating the most pronounced difference (β = 0.31, 95% CI: 0.09-0.53, p = 0.006). This suggests that active viral replication synchronizes inflammatory responses across compartments. Similar patterns were observed for IL-6, IL-10, and IP-10, though effect sizes were more modest. Conversely, neurodegenerative markers (NFL, GFAP, total tau) showed stable plasma-CSF correlations regardless of viral suppression status, supporting their utility as peripheral biomarkers of CNS injury across the spectrum of HIV disease control.

**Fig. 6:**
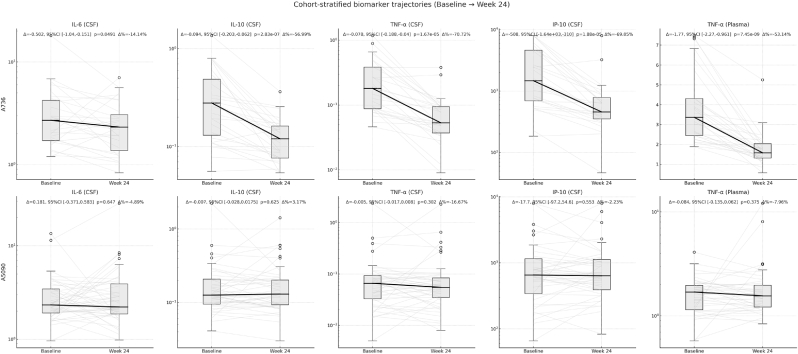
Cohort-stratified biomarker trajectories from baseline to Week 24. CSF TNF-α was a leading correlate of global cognition (NPZ-8) at baseline and after ART. Rows show ACTG 736 (ART-naïve; top) and ACTG 5090 (ART-experienced; bottom). Light gray lines trace individual trajectories, boxplots depict distributions, and bold black lines connect medians. Statistical annotations (Δ, 95% CI, Wilcoxon p, Δ%) are provided for each biomarker. A736 participants showed significant CSF declines in IL-6, IL-10, TNF-α, and IP-10 (all FDR <0.01), with plasma TNF-α also declining (FDR = 0.006). No significant biomarker changes were observed in A5090.

Longitudinal biomarker changes. Longitudinal biomarker trajectories revealed treatment-dependent patterns (Fig. 6). Pooled analyses suggested broad inflammatory declines, but stratified examination revealed that changes were driven entirely by ACTG 736 participants. Among ART-naïve and virally failing participants initiating or switching ART in ACTG A736, CSF inflammatory markers declined substantially over 24 weeks. CSF TNF-α decreased by a median of 22.3% (IQR: −41.2% to −8.1%, p = 0.002), while CSF IL-6, IL-10, and IP-10 showed similar robust declines (all p < 0.01, FDR-corrected). Plasma TNF-α also decreased significantly in this cohort (−18.7%, p = 0.006, FDR-corrected), demonstrating cross-compartment inflammatory resolution following ART initiation. Individual trajectory plots showed consistent downward trends across participants, with minimal variability in response direction. In contrast, ART-experienced participants in ACTG A5090 demonstrated stable biomarker profiles throughout the study period. No inflammatory or neurodegenerative markers showed significant changes from baseline to week 24 (all p > 0.15), with median changes clustering near zero and confidence intervals spanning both positive and negative values. This stability occurred despite randomization to selegiline intervention versus placebo, suggesting that further anti-inflammatory interventions may have a limited impact once viral suppression is achieved. Neurodegenerative markers (NFL, GFAP, tau species, and amyloid peptides) remained stable in both cohorts, indicating that the 24-week observation period was insufficient to detect changes in structural brain injury markers, even in the setting of robust inflammatory improvement.

**Fig. 7:**
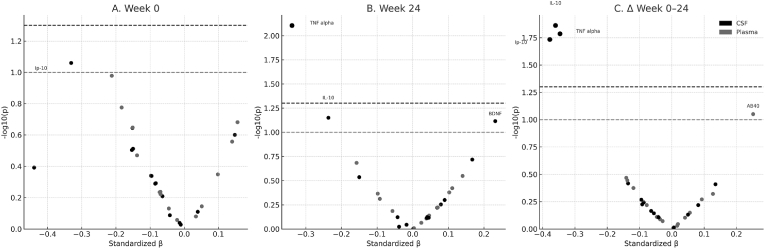
Covariate-adjusted volcano plots showing standardized regression coefficients (β) and –log_10_(p-values) for the associations between individual biomarkers and global neurocognitive performance (NPZ-8). Each point represents one biomarker measured in cerebrospinal fluid (CSF, black) or plasma (gray). Panels represent baseline (A), Week 24 (B), and change scores from baseline to Week 24 (C). Horizontal lines mark uncorrected p-value thresholds (0.05 and 0.10). No associations achieved statistical significance after FDR correction for multiple comparisons. CSF TNF-α at Week 24 showed the strongest nominal association (β = −0.34, p = 0.008, q = 0.19), with longitudinal declines in CSF IL-10, TNF-α, and IP-10 showing similar trends (all p < 0.05, uncorrected).

Individual biomarker associations with cognitive performance (Fig. 7). Volcano plot analysis of covariate-adjusted biomarker-cognition relationships revealed no associations achieving statistical significance after correction for multiple comparisons across the three timepoints examined. At baseline, plasma IL-6 showed the strongest nominal association with poorer cognitive performance (β = −0.21, 95% CI: −0.47-0.05, p = 0.11), while CSF IP-10 demonstrated a similar trend (β = −0.33, 95% CI: −0.71-0.05, p = 0.087). However, these associations had wide confidence intervals, indicating substantial uncertainty. At week 24, CSF TNF-α showed the strongest nominal association with cognitive performance (β = −0.34, 95% CI: −0.59 to −0.09, p = 0.008), ranking above all neurodegeneration markers in effect size magnitude, though this did not survive FDR correction (q = 0.19). Plasma inflammatory markers showed weaker associations at this time point, reinforcing the importance of CNS-specific measurements. No biomarker significantly predicted cognitive change over 24 weeks after FDR correction. The strongest association was between baseline p-Tau217 and ΔNPZ-8 (ρ = −0.12, p = 0.38, q = 0.71), indicating insufficient evidence for predictive relationships within this timeframe. Multivariate models incorporating clinical variables explained <15% of variance in cognitive change scores (R^2^ = 0.12, F = 1.8, p = 0.15).

Longitudinal change analyses revealed that reductions in CSF inflammatory markers tracked nominally with cognitive improvement. Participants showing greater decreases in CSF IL-10 (β = −0.36, p = 0.014), TNF-α (β = −0.35, p = 0.016), and IP-10 (β = −0.38, p = 0.018) demonstrated more favorable cognitive trajectories, though none achieved statistical significance after multiple comparison correction. Notably, changes in neurodegenerative markers (NFL, GFAP, tau species) showed minimal associations with cognitive change, possibly reflecting the relatively short follow-up period. The consistent pattern of CSF TNF-α showing the strongest associations across timepoints, combined with its role in the mediation pathway between HIV replication and cognition, suggests this marker warrants prioritized investigation in future studies with larger sample sizes and longer follow-up periods.

Interaction analyses: GFAP × Tau biomarkers. Complete-case analyses (n = 58– 65) examining GFAP as a modifier of tau biomarker effects yielded null results, with interaction slopes modest (β = +0.08 to +0.41) and global terms nonsignificant (p ≥ 0.56). Exploratory analyses, including all 79 participants, suggested steep GFAP-stratified slopes with significant interactions for t-Tau, p-Tau181, and p-Tau217 (p < 0.001), but these effects were driven by extreme values in high-GFAP individuals with concurrent missing data, producing influential leverage lacking robustness.

Biomarker-cognitive change-score analysis. Change-score interactions among GFAP, Aβ42, and NPZ-8 over 24 weeks (n = 59) showed no significant main or interaction effects (all p > 0.56). Main-effect coefficients were small and non-significant, with the interaction term not differing from zero (β = −0.03, 95% CI -0.42 to +0.36, p = 0.87), indicating that concurrent changes in GFAP and Aβ42 do not jointly predict change in global cognition over 24 weeks.

**Fig. 8:**
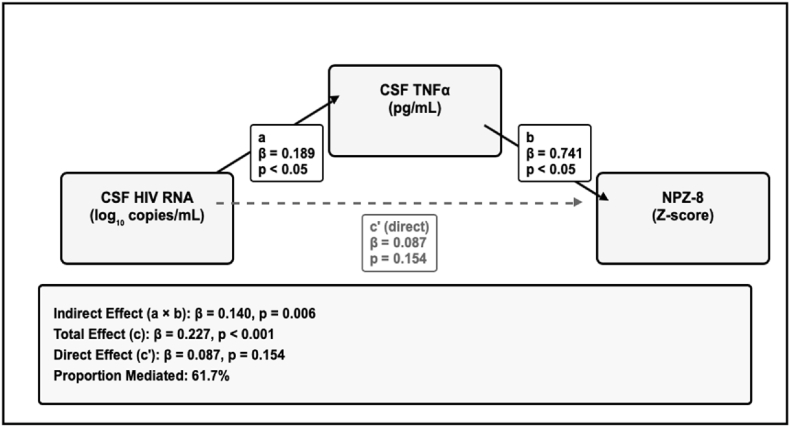
Mediation model testing whether CSF TNF-α mediates the association between CSF HIV RNA and global cognitive performance (NPZ-8). Path coefficients reflect standardized regression estimates. Solid arrows denote statistically significant effects (p < 0.05); dashed arrows are non-significant. The indirect effect (β = 0.14, 95% CI: 0.045–0.235, p = 0.006) accounted for 61.7% of the total effect, indicating that CSF HIV RNA affects cognition primarily via neuroinflammatory pathways. No mediation was observed through plasma TNF-α.

Exploratory Mediation Analysis (Fig. 8). Using bootstrap-based mediation analysis (5000 iterations), we tested whether CSF TNFα mediated the relationship between CSF HIV RNA and cognitive performance. The indirect effect was β = 0.14 (95% CI: 0.045-0.235), suggesting potential mediation, though the direct effect remained marginally significant (p = 0.154). These findings are exploratory and require replication in larger, independent cohorts with prespecified hypotheses.

## 4 Discussion

This study compared CSF and plasma biomarker findings at weeks 0 and 24 from two previously-reported clinical trials (Schiffito et al., 2007; Marra et al., 2009), each of which had primary neurocognitive endpoints. The first, a study of selegiline, found modest cognitive improvement in both the intervention and control arms, with no between-arm differences. The second evaluated the neurocognitive effects of antiretroviral regimens with different CNS-penetration effectiveness (CPE) scores; a differential CPE effect was seen only in individuals who had impaired neurocognitive performance at study entry. Combining both cohorts, CSF biomarkers exhibited strong positive correlations with each other, while plasma biomarker inter-correlations were consistently weaker. These findings are consistent with compartmentalization of inflammation. CSF inflammatory markers declined consistently only in the antiretroviral treatment study, in which viral suppression likely drove these declines.

At baseline, several biomarkers of neurodegeneration (eg, NFL, Aβ42), inflammation (eg, TNF-α) and astrocyte activation (GFAP) showed moderate-to-strong cross-compartment (plasma-CSF) correlations, but these weakened after the interventions, suggesting some biological dynamics, despite the lack of behavioral changes. blood-brain barrier permeability had only a modest influence on cross-compartment correlations. Instead, viral status had a greater influence on cross-compartment relationships for inflammatory markers such that participants with detectable viral loads (those in the antiretroviral intervention study) showed stronger plasma-CSF correlations for inflammatory markers compared to virally suppressed individuals, suggesting that active viral replication synchronizes inflammatory responses across compartments. longitudinal changes in biomarkers were primarily driven by viral suppression status: in the antiretroviral intervention study, reductions in viral load were associated with reductions in inflammatory biomarkers, whereas in the neuroprotective treatment trial, there were no significant changes. By comparison, biomarkers of neurodegeneration did not change significantly in either study.

There was not consistent evidence of associations between biomarker change and cognitive change, though reductions in CSF TNF-α tended to correlate with cognitive improvement. An exploratory mediation analysis further exploring this relationship suggested that TNF-α might mediate the relationship between CSF viral suppression and cognitive performance, but the effect was small.

The most striking pattern emerged from treatment-naïve versus ART-experienced participants. ART-naïve individuals in ACTG 736 demonstrated robust central and peripheral biomarker responses, with consistent CSF declines in TNF-α, IL-6, IL-10, and IP-10, reinforcing these cytokines as sensitive indicators of treatment-related CNS immune modulation. TNF-α showed reproducible declines across both compartments, suggesting it may serve as the most reliable marker of ART-driven inflammatory resolution. The absence of significant changes in ART-experienced ACTG 5090 participants indicates that once systemic viral suppression is established, further treatment with an antioxidant and neuroprotective agent does not measurably reduce inflammatory biomarker levels over 24 weeks.

This study advances the neuro-HIV biomarker field by systematically comparing longitudinal CSF-plasma concordance across viremic and suppressed groups, extending prior cross-sectional reports (Rahimian and He, 2017). The compartment-specific trajectories demonstrate post-ART cytokine decoupling while maintaining persistent GFAP and NFL correlations. Inclusion of p-Tau217 extends biomarker investigation into neurodegenerative processes relevant to aging PWH. Most importantly, our mediation analyses suggest that CSF TNF-α mechanistically links HIV replication to cognitive performance over time, underscoring the necessity of compartment-focused biomarker frameworks.

Limitations: Generalizability to contemporary PWH. This study analyzed archived samples collected between 1999 and 2005, during an era in which ART regimens were largely protease inhibitor or efavirenz-based and rates of incomplete viral suppression were higher than in contemporary practice. These historical features substantially limit direct generalizability to modern PWH populations. Moreover, participants—particularly in ACTG A736—had advanced immunosuppression and high plasma and CSF viral loads. In contrast, current treatment guidelines favor earlier ART initiation, and most individuals in high-resource settings achieve durable viral suppression on integrase strand transfer inhibitor–based regimens. The degree of CNS viral replication and associated inflammatory activation observed here may therefore overestimate what is typically seen in contemporary, early-treated populations. These limitations affect magnitude and clinical applicability more than biological plausibility. The observed compartment-specific coupling between CSF HIV RNA and CSF TNF-α, the tighter inflammatory module within CSF relative to plasma, and the persistence of glial and axonal marker concordance across compartments likely reflect fundamental neuroimmune dynamics. However, whether CSF TNF-α meaningfully mediates cognitive outcomes in virally suppressed, integrase-treated, early-diagnosed PLWH remains unknown. Accordingly, our findings should be interpreted as hypothesis-generating and mechanistic. Replication in contemporary cohorts—with earlier ART initiation, integrase-based regimens, balanced sex representation, and longer follow-up—is necessary before translational or biomarker-guided clinical implications can be considered.

Statistical Limitations. This analysis has several important statistical limitations. First, the relatively small sample sizes (n = 50 and n = 29) provided limited power for detecting modest effect sizes and increased risk of false discoveries despite multiple comparison corrections. Second, the 24-week follow-up period may be insufficient to detect clinically meaningful cognitive changes. Third, many analyses were exploratory rather than hypothesis-driven, and findings require replication in independent cohorts. The mediation analysis, while suggestive, was not prespecified and should be interpreted as hypothesis-generating rather than confirmatory. Across both cohorts, approximately 94% of participants were male; therefore, the results do not generalize to females. Additionally, the demographically corrected norms did not adjust for sex.

In conclusion, these exploratory findings suggest CSF TNF-α warrants further investigation as a correlate of HIV-related neurocognitive impairment; definitive conclusions await replication in larger, adequately powered studies with prespecified hypotheses. The results refine the role of plasma GFAP and NFL as CNS surrogates, and underscore the importance of prespecified, compartment-specific analytic strategies. The persistent null findings for cognitive prediction highlight the complexity of neurocognitive change in treated HIV infection and suggest longer follow-up periods with more sensitive cognitive measures may be required. Future work should validate our findings in larger cohorts concerning TNF-α and cognition, as anti-TNF-α therapies are available and could be considered as interventions to improve cognition in PWH.

## CRediT authorship contribution statement

**Ronald J. Ellis:** Writing – original draft, Visualization, Conceptualization. **Yajing Bao:** Writing – review & editing, Formal analysis, Data curation, Conceptualization. **Huichao Chen:** Writing – review & editing, Formal analysis, Data curation. **Scott Letendre:** Writing – review & editing. **Ahmed Chenna:** Writing – review & editing, Resources. **Brandon Yee:** Writing – review & editing, Methodology. **John Winslow:** Writing – review & editing. **Christos Petropoulos:** Writing – review & editing. **Shelli Farhadian:** Writing – review & editing. **Shibani S. Mukerji:** Writing – review & editing. **Albert Anderson:** Conceptualization.

## Declaration of competing interest

The authors declare no conflicts of interest.

## Data Availability

Data will be made available on request.
