# Supplementary material for: Linking neuroinflammation and neurodegeneration to cognitive decline in HIV
Source: Brain Behav Immun Health. 2026 Apr 14;54:101241. doi: 10.1016/j.bbih.2026.101241 (PMC13098446; doi:10.1016/j.bbih.2026.101241)
Supplement: Multimedia component 1 [file mmc1.docx]

# Supplemental Table

| **Supplemental Table 1**. Neuropsychological tests used and the domains they represent. | | |
| --- | --- | --- |
| **Test** | **ACTG 736 Domain(s)** | **ACTG 5090 Domain(s)** |
| Timed Gait | Gross motor speed, balance | Gross motor speed, balance |
| Grooved Pegboard (Dominant) | Fine motor dexterity, psychomotor speed | Fine motor dexterity, psychomotor speed |
| Grooved Pegboard (Non-Dominant) | Fine motor dexterity, psychomotor speed | Fine motor dexterity, psychomotor speed |
| Trail Making Test Part A | Attention, processing speed | Attention, processing speed |
| Trail Making Test Part B | Executive function (set-shifting), processing speed | Executive function (set-shifting), processing speed |
| **Digit Symbol** | Processing speed, complex attention |  |
| **Symbol Digit** |  | Processing speed, complex attention |
| **Finger Tapping** **(Dominant)** | Motor speed |  |
| **Finger Tapping (Non-Dominant)** | Motor speed |  |
| **Choice Reaction Time** |  | Psychomotor speed, sustained attention |
| **Sequential Reaction Time** |  | Psychomotor speed, motor learning |
| *Tests in bold differed between the two studies. | | |
